# Differential Cerebral Cortex Transcriptomes of Baboon Neonates Consuming Moderate and High Docosahexaenoic Acid Formulas

**DOI:** 10.1371/journal.pone.0000370

**Published:** 2007-04-11

**Authors:** Kumar S.D. Kothapalli, Joshua C. Anthony, Bruce S. Pan, Andrea T. Hsieh, Peter W. Nathanielsz, J. Thomas Brenna

**Affiliations:** 1 Division of Nutritional Sciences, Cornell University, Savage Hall, Ithaca, New York, United States of America; 2 Mead Johnson and Company, Evansville, Indiana, United States of America; 3 Center for Pregnancy and Newborn Research, University of Texas Health Science Center, San Antonio, Texas, United States of America; University of Massachusetts, United States of America

## Abstract

**Background:**

Docosahexaenoic acid (DHA, 22:6n-3) and arachidonic acid (ARA, 20:4n-6) are the major long chain polyunsaturated fatty acids (LCPUFA) of the central nervous system (CNS). These nutrients are present in most infant formulas at modest levels, intended to support visual and neural development. There are no investigations in primates of the biological consequences of dietary DHA at levels above those present in formulas but within normal breastmilk levels.

**Methods and Findings:**

Twelve baboons were divided into three formula groups: Control, with no DHA-ARA; “L”, LCPUFA, with 0.33%DHA-0.67%ARA; “L3”, LCPUFA, with 1.00%DHA-0.67%ARA. All the samples are from the precentral gyrus of cerebral cortex brain regions. At 12 weeks of age, changes in gene expression were detected in 1,108 of 54,000 probe sets (2.05%), with most showing <2-fold change. Gene ontology analysis assigns them to diverse biological functions, notably lipid metabolism and transport, G-protein and signal transduction, development, visual perception, cytoskeleton, peptidases, stress response, transcription regulation, and 400 transcripts having no defined function. *PLA2G6*, a phospholipase recently associated with infantile neuroaxonal dystrophy, was downregulated in both LCPUFA groups. *ELOVL5*, a PUFA elongase, was the only LCPUFA biosynthetic enzyme that was differentially expressed. Mitochondrial fatty acid carrier, *CPT2*, was among several genes associated with mitochondrial fatty acid oxidation to be downregulated by high DHA, while the mitochondrial proton carrier, *UCP2*, was upregulated. *TIMM8A*, also known as deafness/dystonia peptide 1, was among several differentially expressed neural development genes. *LUM and TIMP3*, associated with corneal structure and age-related macular degeneration, respectively, were among visual perception genes influenced by LCPUFA. *TIA1*, a silencer of *COX2* gene translation, is upregulated by high DHA. Ingenuity pathway analysis identified a highly significant nervous system network, with epidermal growth factor receptor *(EGFR)* as the outstanding interaction partner.

**Conclusions:**

These data indicate that LCPUFA concentrations within the normal range of human breastmilk induce global changes in gene expression across a wide array of processes, in addition to changes in visual and neural function normally associated with formula LCPUFA.

## Introduction

The vertebrate central nervous system (CNS) is rich in the long chain polyunsaturated fatty acids (LCPUFA) docosahexaenoic acid (DHA) and arachidonic acid (ARA), and this composition is highly conserved across species[1]. Within the CNS, DHA and ARA are found at highest concentration in gray matter[2], and DHA is particularly concentrated in retinal photoreceptor membranes where it has long been known to play a key role in visual excitation[3]. In humans, DHA and ARA accumulate perinatally[4] and many studies of DHA/ARA supplemented formula show improvements in visual acuity[5] and cognitive function[6].

Despite the high demand for LCPUFA during perinatal CNS development, the best current evidence indicates that ARA and DHA can be synthesized only very inefficiently from dietary precursors and must be obtained from the diet[7]. DHA and ARA are present in all human milks studied to date[8], however their concentration is variable. For DHA it is closely linked to the mother's intake of preformed DHA, which is in turn reflective of the mother's intake of fatty fish or fish/marine oil supplements[9], [10], [11], [12]. Dietary factors associated with ARA are less well understood[13]. High levels of precursor fatty acids LA and ALA in formulas yield negligible or at most moderate increases in plasma ARA and DHA concentrations[14], [15]. However, in randomized controlled studies where preterm and term infants are fed preformed DHA and ARA supplemented formula, improvements in LCPUFA status as well as cognitive development and visual functions are observed [16], [17], [18], [19], [20].

While the importance of LCPUFA in infant nutrition has been established, the underlying mechanisms are only beginning to be understood. Brain accretion of LCPUFA is most intense during the brain growth spurt in the third trimester of pregnancy and during early childhood[21], [22], [23], [24]. Selective incorporation and functional properties of LCPUFA, especially DHA, in retinal and neural membranes suggests a specific role in the modulation of protein-lipid interactions, membrane bound receptor function, membrane permeability, cell signaling, regulation of gene expression and neuronal growth [25], [26], [27], [28], [29], [30]. Additionally, LCPUFA mediate metacrine regulation and changes in gene expression by interacting with nutrient sensitive transcription factors [18], [31]. Accordingly, poor nutrition during prenatal life and early infancy may have a lasting influence on neural function, as well as adult risk for chronic diseases [32], [33], [34]. Studies suggest that infant diets low in LCPUFA can lead to health complications such as insulin resistance, obesity, or blood pressure changes later in life [35], [36].

DHA and ARA were introduced in 2002 to infant formulas in the United States, but initial concentrations varied over more than a factor of two (range of DHA 8-19 mg/kcal; ARA 21-34 mg/kcal),[37] and there are no dose response studies in humans or non-human primates available as a guide to optimal levels. A previous study in our laboratory on 4-week-old baboon neonates with preformed DHA and ARA (0.33%,w/w DHA and 0.67% ARA) in formulas showed DHA concentrations in various regions of the brain similar to breastfed controls, with the important exception of the cerebral cortex; ARA concentrations were not much altered by inclusion of dietary preformed ARA[2]. These results inspired our present study on 12 week old baboon neonates with the higher level of 1.00% DHA, along with 0.67% ARA. We report elsewhere [38] that DHA in the precentral gyrus of cerebral cortex increased beyond that achieved for 0.33% DHA, while regions such as the basal ganglia that reached DHA concentrations similar to breastfed animals at 0.33% DHA did not show further increases with 1.00% DHA. These data demonstrate that formula DHA in the high normal range of breastmilk DHA supports enhanced cortex DHA, but do not reveal how this compositional change may influence metabolic function.

To gather mechanistic information on the role of DHA and ARA in the primate cerebral cortex, we investigated global gene expression for cerebral cortex of animals in this study, consuming two different levels of formula DHA both within the range found in human breastmilk[8]. We report here changes in expression of thousands of genes in 12-week-old baboons in response to two different levels of LCPUFA: 0.33%DHA and 0.67% ARA; 1.00% DHA and 0.67% ARA. We have reported in detail on consequences for tissue fatty acid composition [38] and other factors elsewhere (Hsieh et al., 2007, submitted).

## Results and Discussion

Significance analysis (*P*<0.05) identified changes in expression levels of 1108 probe sets (ps) for comparisons of L3/C and/or L/C, representing 2.05% of the total>54,000 ps on the oligoarray. Most ps showed <2-fold change. For the L/C comparisons, 534 ps were upregulated, and 574 ps were downregulated, while for the L3/C comparisons, 666 ps were upregulated and 442 ps were downregulated, showing that more genes were upregulated in the cerebral cortex in response to increasing formula ARA and DHA. Functional characterization by gene ontology of these differentially regulated genes assigns them to diverse biological processes including lipid and other metabolism, ion channel and transport, development, visual perception, G-protein and signal transduction, regulation of transcription, cell cycle, cell proliferation, apoptosis etc. Known functions were assigned to 702 differentially expressed probe sets, whereas 406 ps had no known functions as shown in Table S1A, S1B, S1C, S1D. Probe sets with ≥1.4 fold expression change are presented in Table S2. Experimental details for nine genes used for confirmatory RT-PCR analysis are presented in Table S3. We note that in our L/C and L3/C comparisons, expression patterns fall into four groups, L/C and L3/C both upregulated and both downregulated, or one upregulated and one downregulated. Because the L and L3 groups have the same amount of ARA but different amounts of DHA, our treatments do not strictly represent a DHA dose response. The L/C comparison corresponds to inclusion of DHA and ARA at current levels near the worldwide breastmilk means, while the L3 group corresponds to DHA near the worldwide high [8].

Nine genes were tested by quantitative real time PCR to confirm the array results, as shown in Table S4. All were qualitatively consistent with the gene array results.

We highlight results in several categories of gene ontogeny as follows.

### Lipid (fatty acid and cholesterol) Metabolism

Table 1 presents results from genes related to lipid metabolism that are regulated by dietary LCPUFA.

**Table 1:** Lipid and energy metabolism gene fold-changes in expression profiles.

Metabolism	Gene Symbol	Unigene ID	L	L3
Lipid	*ATP8B1*	Hs.569910	1.28	1.36
	*PDE3A*	Hs.386791	1.08	1.30
	*ELOVL5*	Hs.520189	−1.02	1.11
	*ACSL3*	Hs.471461	−1.13	1.08
	*HNF4A*	Hs.116462	1.06	−1.16
	*CLPS*	Hs.1340	1.02	−1.16
	*ALDH3B2*	Hs.87539	1.05	−1.16
	*PLCE1*	Hs.20022	−1.10	−1.19
Fatty acid oxidation	*ACADSB*	Hs.81934	−1.10	1.38
	*ACAD10*	Hs.331141	−1.08	1.10
	*GLYAT*	Hs.274336	1.01	1.30
	*ADH5*	Hs. 78989	1.03	1.22
	*CPT2*	Hs.145384	1.10	−1.22
Energy	*LEP*	Hs.194236	−1.01	1.17
Ceramide	*NSMAF*	Hs.372000	−1.04	1.31
	*LASS5*	Hs.270525	1.06	1.11
Glycosphingolipid	*SPTLC2*	Hs.435661	1.27	1.40
Steroid	*OSBP2*	Hs.517546	−1.17	1.35
	*UGT2B15*	Hs.150207	1.04	1.21
	*SULT2B1*	Hs.369331	1.04	−1.38
Phospholipid	*DGKE*	Hs.546318	−1.10	1.17
	*PLA2G6*	Hs.170479	−1.09	−1.20
Prostaglandin and Leukotriene	*TEBP*	Hs.50425	1.02	1.52
	*ANXA3*	Hs.480042	1.26	−1.04
	*LTC4S*	Hs.456	−1.33	−1.24
Cholesterol	*DHCR24*	Hs.498727	−1.18	1.17
	*PRKAG2*	Hs.131133	−1.07	1.09
	*PRKAA1*	Hs.43322	1.09	−1.02
	*SOAT1*	Hs.496383	−1.09	−1.12
	*FDFT1*	Hs.546253	1.01	−1.13

Genes related to phospholipids biosynthesis (*PLA2G6* and *DGKE*) were differentially expressed. *PLA2G6* was downregulated in both groups. This gene codes for the Ca-independent cytosolic phospholipase A2 Group VI. Alterations in this gene have very recently been implicated as a common feature of neurodegenerative disorders involving iron accumulation [39], as well as the underlying factor in infantile neuroaxonal dystrophy, a neurodegenerative disorder caused by accumulation of iron in the globus pallidus and resulting in death by age 10[40]. In a previous study of four week old breastfed baboons, the globus pallidus was found to have 15.8±0.5% DHA (w/w of total fatty acids) and was the richest in DHA of 26 CNS regions examined[2]. The globus pallidus is also rich in ARA, with 10.3% (w/w) in four week old baboons. PLA2 are a superfamily of enzymes that liberate fatty acids from the sn-2 position of phospholipids; in the globus pallidus DHA and ARA are the most abundant acyl groups at this site.

Remarkably, among the elongation and desaturation enzymes associated with LCPUFA synthesis, only a single elongation enzyme was differentially expressed. The human *ELOVL5* transcript was downregulated slightly in the L/C group and upregulated in the L3/C group. This enzyme, also called *HELO1*, catalyzes the two carbon elongation of polyunsaturated 18 and 20 carbon fatty acids [41], [42].

We also found that *DGKE* was upregulated in the L3/C comparison. Genes involved in ceramide metabolism (*NSMAF, LASS5*), glycosphingolipid metabolism (*SPTLC2*) and steroid metabolism (*OSBP2, UGT2B15*) showed increased expression in L3/C group, whereas *NSMAF* and *OSBP2* were downregulated in L/C group.

The best studied role of ARA is as a precursor for eicosanoids including prostaglandins, leukotrienes, and thromboxanes. One of the genes derived from membrane-bound ARA, which catalyze the first step in the biosynthesis of cysteinyl leukotrienes, Leukotriene C4 synthase (*LTC4S*), is downregulated in both DHA-ARA groups. *LTC4S* is a potent proinflammatory and anaphylactic mediator [43]. An elevated level of mRNA for *PGES3* (prostaglandin E synthase 3) was observed in both the groups. *PGES3* is also known as *TEBP* (telomerase-binding protein p23) or inactive progesterone receptor, 23-KD (*p23*). *p23*, a ubiquitous highly conserved protein which functions as a co-chaperone for the heat shock protein, *HSP90*, participates in the folding of a number of cell regulatory proteins [44], [45]. *p23* has been demonstrated to bind to human telomerase reverse transcriptase (hTERT) and contribute to telomerase activity [46]. Decreased levels of Annexin A3 (*ANXA3*) also known as Lipocortin III was observed with increasing DHA.

Genes involved in fatty acid oxidation (*ACADSB, ACAD10* and *GLYAT*) were upregulated, and carnitine palmitoyltransferase II (*CPT2*) downregulated, in the L3/C group. ACADs (acyl-CoA dehydrogenases) are a family of mitochondrial matrix flavoproteins that catalyze the dehydrogenation of acyl-CoA derivatives and are involved in the β-oxidation and branched chain amino-acid metabolism [47], [48]. Both the ACADs family members *ACADSB* and *ACAD10* were upregulated in L3/C group, consistent with greater energy production in the high DHA group. Mitochondrial-specific *GLYAT* (glycine-N-acyltransferase) also known as acyl CoA:glycine N-acyl transferase (*ACGNAT*), conjugates glycine with acyl-CoA and participates in detoxification of various drugs and xenobiotics [49], [50]. Mawal et al [50] suggested that delayed development of *GLYAT* might impair detoxification process in children.

Genes involved in cholesterol biosynthesis, *DHCR24, PRKAG2, PRKAA1, SOAT1*, and *FDFT1* showed significant associations with LCPUFA levels. Increasing DHA upregulated *DHCR24* and *PRKAG2*, downregulated *PRKAA1, SOAT1* and *FDFT1. DHCR24* (24-dehydrocholesterol reductase) also known as selective AD indicator 1 (*SELADIN1*) catalyzes the reduction of the delta-24 double bond of sterol intermediates during cholesterol biosynthesis [51]. *SELADIN1* may activate estrogen receptor in the brain and protect from beta-amyloid-mediated toxicity [52]. Decreased expression of *SELADIN1* is observed in brain regions of patients with Alzheimer's disease [53]. *PRKAG2* (protein kinase, AMP-activated, gamma 2) is a member of AMP-activated protein kinase (AMPK) family. AMPKs perform multifunctional roles in calcium signaling, weight loss, regulation of energy metabolism in heart [54], [55], [56].

*SOAT1* (sterol O-acyl transferase) or Acyl-coenzyme A: cholesterol acyl transferase (*ACAT*) is an intracellular protein which catalyzes the formation of cholesterol esters in endoplasmic reticulum and is involved in lipid droplets that are characteristic of foam cells of atherosclerotic plaques [57], [58], [59].

Increased expression was detected for *ATP8B1*, *PDE3A* in both groups, comparatively more in L3/C, while transcripts involving *HNF4A* (Hepatic nuclear factor-4α), *CLPS* and *ALDH3B2* showed decreased expression with increasing DHA. Intrahepatic cholestasis, or impairment of bile flow, is an important manifestation of inherited and acquired liver disease resulting in hepatic accumulation of the toxic bile acids and progressive liver damage. Bile acids enhance efficient digestion and absorption of dietary fats and fat-soluble vitamins, and are the main route for excretion of sterols. Expression of *ATP8B1* is high in the small intestine, and mutations in *ATP8B1* gene have been linked to intrahepatic cholestasis [60], [61]. *ATP8B1* expression was confirmed by real time PCR (Table S4). *PDE3A* (phosphodiesterase 3A, cGMP-inhibited) is a 120 kDa protein found in myocardium and platelets [62]. Ding et al[63] showed significantly decreased expression of *PDE3A* in the left ventricles of failing human hearts. *PDE3A* expression is required for the regulation of penile erection in humans [64].

Leptin (*LEP*), which has a role in energy metabolism, was upregulated in L3/C group. Leptin is a secreted adipocyte hormone that plays a pivotal role in the regulation of food intake and energy homeostasis [65], [66]. Leptin suppresses feeding and decreases adiposity in part by inhibiting hypothalamic Neuropeptide Y synthesis and secretion [67], [68].

### Ion Channel and Transport

Expression levels of transcripts involved in ion channel and transporter activity were altered by dietary LCPUFA (Table S5). Uncoupling protein 2, *LOC131873* (hypothetical protein) and *ATP11C*, which have ion channel activity, are upregulated in both the groups but moreso in L3/C. Other transcripts with ion channel activity, including *VDAC3, FTH1, KCNK3, KCNH7* and *TRPM1* were upregulated in L3/C group and downregulated in L/C. *GLRA2, TRPV2* and *HFE* are upregulated in L/C and repressed in L3/C. *P2RX2, GRIA1* and *CACNA1S* are repressed in both the groups.

One of our significant observations is the increased expression of uncoupling protein 2 (*UCP2*), a mitochondrial, proton carrier. Our data shows, for the first time, increased expression of *UCP2* in neonatal cerebral cortex associated with dietary LCPUFA; increased expression is observed in both the groups but more in L3/C. QRT-PCR confirmed the array results (Table S4). Nutritional regulation and induction of mitochondrial uncoupling proteins resulting from dietary n3-PUFA in skeletal muscle and white adipose tissue have been observed [69], [70]. Increased *UCP2* expression is beneficial in diseases associated with neurodegeneration, cardiovascular and type 2 diabetes[71]. Dietary fats in milk increased the expression and function of *UCP2* in neonatal brain and protected neurons from excitotoxicity [72].

*VDAC3* (voltage-dependent anion channel 3) belongs to a group of pore forming proteins found in the outer mitochondrial membrane and in brain synaptic membranes [73], [74]. Massa et al [75] observed a significant reduction of *VDAC3* mRNA levels in the skeletal muscle and brains of dystrophin-deficient mdx mice during postnatal development. Mice lacking *VDAC3* exhibit infertility [76]. All the transcripts (*VDAC3, KCNK3* and *KCNH7*) having voltage-gated anion channel porin activity were upregulated with increasing DHA. *FTH1* (Ferritin heavy chain 1) is required for iron homeostasis and it has been previously shown to be expressed in human brain [77].

Genes encoding small molecule transporters were differentially expressed, including carriers of glucose (*SLC2A1, SLC5A4*), chloride (*SLC12A6*), sodium (*SLC13A3*), monoamine (*SLC18A2*) and others (*SLC26A4, SLC17A6*). These transporters might help in exchange of nutrients and metabolites. Members of the cytochrome P and B family of proteins were also differentially expressed. Transcripts encoding *VDP, RSAFD1, C1QG* and *OXA1L* were significantly repressed by increasing DHA.

### G-Proteins and Signaling

Numerous genes encoding G-protein activity were differentially regulated (Table S5), and the majority were induced by high DHA. *GNA13, GNA14, PTHR2, RCP9* and *FZD3* showed increased expression in both DHA groups. *EDG7, SH3TC2, GNRHR, ADRA1A, BLR1, GPR101, GPR20* and *OR8G2* were downregulated in L/C and upregulated in L3/C. *NPY1R* is downregulated in both the groups.

DHA regulates G-protein signaling in the brain and retina [78]. G-proteins are membrane-associated proteins which promote exchange of GTP for GDP and regulate signal transduction and membrane traffic[79]. *GNA13* deficiency impairs angiogenesis in mice [80] while *GNA14* activates the NF-κB signaling cascade [81]. Parathyroid hormone receptor 2 (*PTHR2*) is activated by parathyroid hormone and is relatively abundant in the CNS [82], [83]. *RCP9*, also known as calcitonin gene-related peptide-receptor component protein, may have a role during hematopoiesis [84]. Tissir and Goffinet [85] showed expression of *FZD3* during postnatal CNS development in mice. *FZD3* array results were confirmed by SYBR green real time PCR assay (Table S4).

Neuropeptide Y is a 36-amino acid peptide with strong orexigenic effects *in vivo* [86]. Two major subtypes of NPY (Y1 and Y2) have been defined by pharmacologic criteria. *NPY1R* was suggested to be unique for the control of feeding [87]. Pedrazzini et al [88] observed a moderate but significant decrease in food intake in mice lacking the *NPY1R* gene.

*EDG7* (endothelial differentiation, lysophosphatidic acid G-protein-coupled receptor, 7) mediates calcium mobilization[89]. Mutation in the *SH3TC2* gene causes childhood-onset of a neurodegenerative disorder affecting motor and sensory neurons[90].

Several signaling proteins (*NF1, WSB1, SOCS4, RIT1, CD8B1, OR2A9P* and *RERG*) were upregulated in both groups. Genes that are upregulated in L3/C and downregulated in L/C were also observed, specifically *PDE4D, KRAS, ITGA2, PLCXD3, WNT8A, ARHGAP4, RAPGEF6, OR2F1/OR2F2, CCM1* and *SFRP2*, while a few genes (*WNT10A*, *ADCY2, OGT, DDAH1* and *BCL9*) were upregulated in L/C and downregulated in L3/C. *IQGAP3, GCGR, APLN, CYTL1, GRP, LPHN3, CNR1, VAV3* and *MCF2* were downregulated in both the groups (Table S5).

*NF1* is a tumor-suppressor gene; mutations in this gene cause neurocutaneous defects [91]. *NF1* gene expression and function are needed for normal fracture healing [92]. *NF1* expression levels were confirmed by QRT-PCR (Table S4). *WSB1* is a SOCS-box-containing WD-40 protein expressed during embryonic development in chicken [93]. RAS and RAS related gene families of small GTPases (*RIT1, KRAS, RERG* and *RAPGEF6*) were upregulated by increasing DHA.

Diets deficient in n-3 PUFA induce substitution of n-6 DPA (22:5n-6) in neural membranes, and impairment of functions mediated by G protein mediated signaling, such as visual perception, learning and memory, and olfactory discrimination. Abundant evidence indicates that this results in reduced rhodopsin activation, and signaling in rod outer segments compared to DHA-replete animals[78], [94], [95], [96], [97].

### Development

Table 2 shows differential expression of 24 genes related to development. The products of 11 transcripts play a role in nervous system development. The expression of *TIMM8A, NRG1, SEMA3D* and *NUMB* genes were upregulated in both L/C and L3/C groups. *HES1* and *SIM1* were downregulated in both the groups. *GDF11, SMA3/SMA5, SH3GL3* were downregulated in L/C and upregulated in L3/C. The mRNA levels of growth factors *FGF5* and *FGF14* displayed increased abundance in L/C and decreased abundance in L3/C.

**Table 2:** Development gene fold-changes in expression profiles.

Development	Gene Symbol	Unigene ID	L	L3
Nervous system	*TIMM8A*	Hs.447877	1.04	1.57
	*NRG1*	Hs.453951	1.02	1.21
	*SEMA3D*	Hs.201340	1.10	1.14
	*NUMB*	Hs.585653	1.01	1.10
	*HES1*	Hs.250666	−1.30	−1.63
	*SIM1*	Hs.520293	−1.16	−1.16
	*GDF11*	Hs.591023	−1.18	1.09
	*SMA3///SMA5*	Hs.482414/484969/588240	−1.08	1.06
	*SH3GL3*	Hs.270055/458285	−1.16	1.04
	*FGF5*	Hs.37055	1.08	−1.20
	*FGF14*	Hs.591206	1.01	−1.10
Muscle	C6orf97	Hs.130239	−1.03	1.34
	*CALD1*	Hs.490203	1.09	1.14
Skeletal	*BAPX1*	Hs.105941	1.05	1.08
Heart	*GATA4*	Hs.243987	−1.02	1.22
Epidermis	*S100A7*	Hs.112408	−1.06	1.27
	*FGF7*	Hs.122006	1.14	1.02
	*SCEL*	Hs.115166	−1.01	−1.13
Ectoderm/Mesoderm	*SMURF1*	Hs.189329	1.15	1.32
	*TCF21*	Hs.78061	−1.12	−1.18
Gametogenesis	*OTEX*	Hs.196956	1.09	1.24
	*TCP11*	Hs.435371	−1.02	1.08
	*CDV1*	Hs.528382	−1.001	−1.10
	*SPAG6*	Hs.527698	−1.03	−1.22

*TIMM8A* also known as Deafness/Dystonia Peptide 1 (*DDP1*) is a well conserved protein organized in mitochondrial intermembrane space. Loss-of-Function mutations in the *TIMM8A* gene cause Mohr-Tranebjaerg syndrome (a progressive neurodegenerative disorder with deafness, blindness, dystonia and mental deficiency) and Jensen syndrome (opticoacoustic nerve atrophy with dementia) [98], [99], [100]. TaqMan assay confirmed the array results (Table S4). *NRG1* is essential for the development and function of the CNS facilitating the neuronal migration and axon guidance [101], [102]. *NUMB* negatively regulates notch signaling and plays a role in retinal neurogenesis, influencing the proliferation and differentiation of retinal progenitors and maturation of postmitotic neurons [103]. *HES1* (Hairy/Enhancer of Split, Drosophila, Homolog of, 1) a basic helix-loop-helix protein is downregulated. Decreased expression of *HES1* is observed as neurogenesis proceeds and in case of persistent expression differentiation of neuronal cells are blocked in the CNS [104].

### Visual Perception

Nine transcripts having a role in visual perception were differentially expressed (Table 3). Genes coding for *LUM, EML2, TIMP3* and *TTC8* were upregulated in both the supplement groups. *IMPG1* was upregulated in L3/C and downregulated in L/C. *RGS16* and *TULP2* were upregulated in L/C and downregulated in L3/C. *RAX* and *IMPDH1* were downregulated in both the supplement groups.

**Table 3:** Visual perception gene fold-changes in expression profiles.

Gene Product	Unigene ID	L	L3
Lumican (*LUM*)	Hs.406475	1.03	1.30
Interphotoreceptor matrix proteoglycan 1 (*IMPG1*)	Hs.590893	−1.03	1.18
Echinoderm microtubule associated protein like 2 (*EML2*)	Hs.24178	1.07	1.15
TIMP metallopeptidase inhibitor 3 (*TIMP3*)	Hs.297324	1.28	1.05
Tetratricopeptide repeat domain 8 (*TTC8*)	Hs.303055	1.10	1.01
IMP (inosine monophosphate) dehydrogenase 1 (*IMPDH1*)	Hs.534808	−1.20	−1.12
Tubby like protein 2 (*TULP2*)	Hs.104636	1.07	−1.15
Retina and anterior neural fold homeobox (*RAX*)	Hs.278957	−1.10	−1.24
Regulator of G-protein signalling 16 (*RGS16*)	Hs.413297	1.01	−1.26

Lumican (*LUM*), is an extracellular matrix glycoprotein and a member of the small-leucine-rich-proteoglycan (SLRP) family [105]. It is widely distributed in the corneal stoma and connective tissues [106]. Lumican helps in the establishment of corneal stromal matrix organization during neonatal development in mice. Those lacking lumican exhibit several corneal related defects [107]. It is important for corneal transparency in mice [108]. TaqMan assay showed 5-fold more upregulation of *LUM* more than the microarray data (Table S4). Mutations in *TIMP3* gene result in autosomal dominant disorder Sorsby's fundus dystrophy an age-related macular degeneration of retina [109]. Clarke et al [110] suggested that a possible mechanism for retinal degeneration in Sorsby's fundus dystrophy was traceable to nutrition.

*IMPG1* is a proteoglycan which participates in retinal adhesion and photoreceptor survival [111]. Higher amounts of DHA in the infant formula increased the expression of *IMPG1*. Expression of *RAX* transcript is decreased in both the supplement groups. Increased *RAX* expression is seen in the retinal progenitor cells during the vertebrate eye development and is downregulated in the differentiated neurons[112], [113]. DHA is well known to promote neurite growth in the brain [30]; this could be the possible reason for *RAX* downregulation in our study.

### Integral to Membrane/Membrane Fraction

Transcripts that are integral part of biological membranes or within the membrane fractions were differentially expressed (Table S5). *EVER1, PERP, Cep192, SSFA2, LPAL2, TMEM20, TM6SF1* were upregulated in both the groups. *ORMDL3, SEZ6L, HYDIN, TA-LRRP, PKD1L1* were upregulated in L3/C and downregulated in L/C. *MFAP3L* was upregulated in L/C and downregulated in L3/C. Transcripts of *GP2* and *SYNGR2* were downregulated in both the groups.

Numbers of transcripts were upregulated by increased DHA in the formulas. LCPUFA can affect biological membrane functions by influencing membrane composition and permeability, interaction with membrane proteins, membrane-bound receptor function, photoreceptor signal transduction and transport [114], [115], [116]. Mutations in *EVER1* or transmembrane channel-like 6 (*TMC6*) gene cause epidermodysplasia verruciformis, a type of skin disorder [117]. *HYDIN* is a novel gene and nearly-complete loss of its function due to mutations causes congenital hydrocephalus in mice [118]. The exact function of *GP2* is unknown, but it has been associated with the secretory granules in the pancreas [119].

### Programmed Cell Death/Apoptosis

Transcripts with apoptotic activity were differentially expressed (Table S5). Seven out of nine transcripts in our study were upregulated with increasing DHA, including *CARD6, TIA1, BNIP1, FAF1, GULP1, CASP9* and *FLJ13491*. Programmed cell death (PCD) plays an important role during the development of immune and nervous systems [120]. Jacobson et al [121] proposed PCD as an important event in eliminating unwanted cells during development. Mice with targeted deletion of *CASP3* die perinatally due to vast excesses of cells deposition in their CNS as a result of decreased apoptotic activity [120]. *CARD6* (caspase recruitment domain protein 6) is upregulated in both the groups. It is a microtubule-interacting protein that activates NF-КB and takes part in the signaling events leading to apoptosis [122]. *TIA1* is upregulated in L3/C and downregulated in L/C. *TIA1* is a member of RNA-binding protein family with pro-apoptotic activity, and it silences the translation of cyclooxygenase-2 (*COX2*). Narayanan et al, [123] suggested that DHA indirectly increases the expression of genes which downregulate *COX2* expression. The *COX2* enzyme catalyzes the rate-limiting step for prostaglandin production, which influence many processes including inflammation [124]. Downregulation of *TIA1* in L/C could be due to the influence of ARA, the major *COX2* substrate, rather than that of DHA which is a competitive inhibitor. *GULP1* assists in efficient removal of the apoptotic cells by phagocytosis [125]. *CASP9* activates caspase activation cascade and is an important component of mitochondrial apoptotic pathway [126].

### Cytoskeleton and Cell adhesion

Dietary LCPUFA regulated expression of several transcripts involved in cytoskeleton and cell adhesion (Table S5). The expression of 27 ps involved in cytoskeleton was altered. *MYO1A* and *MYO5A* were upregulated with increasing amounts of DHA whereas *MYO1E* showed decreased expression. Myosin-1 isoforms are membrane associated molecular motors which play essential roles in membrane dynamics, cytoskeletal structure and signal transduction [127]. *COL4A6* and *COL9A3* showed increased expression whereas *COL4A2* and *COL9A2* showed decreased expression with increasing DHA. Type IV collagen is the major component of the basement membrane. Mild forms of Alport nephropathy is associated with deletion in *COL4A6* gene [128] and eye abnormalities are common in people afflicted with Alport syndrome [129]. *WASL*, also known as neural WASP (*WASP*), was upregulated in both the groups. Actin cytoskeleton regulation is vital for brain development and function. *WASL* is an actin-regulating protein and mediates filopodium formation [130], [131], [132]. *HIP1* (huntingtin interacting protein 1) and *HOOK2* (hook homolog 2) were downregulated in both the groups.

The expression levels of 15 transcripts involved in cell adhesion changed as a result of dietary LCPUFA (Table S5). *BTBD9, CD44, ARMC4, CD58, LOC389722* and *PCDHB13* showed increased expression in both the groups. Glycoprotein *CD44* is a cell-surface adhesion molecule that is involved in cell-cell and cell-matrix interactions [133] while *PCDHB13* is a member of protocadherin beta family of transmembrane glycoproteins [134]. *NLGN3* and *CYR61* were downregulated in both groups.

### Peptidases

Several transcripts having peptidase activity were differentially expressed (Table S5). *SERPINB6* is significantly upregulated in L3/C and downregulated in L/C. Of note, the ADAM families of proteins (*ADAM17, ADAM33*, and *ADAMTS16*) were upregulated and *ADAMTS15* was downregulated in both the supplement groups. ADAM proteins are membrane-anchored glycoproteins named for two of the motifs they carry: an adhesive domain (disintegrin) and a degradative domain (metalloprotease) [135]. These proteins are involved in several biological processes including cell-cell interactions, heart development, neurogenesis and muscle development [136], [137], [138], [139]. *ADAM17* is required for proteolytic processing of other proteins and have been reported to participate in cleaving of the amyloid precursor protein [140], [141]. Loss of *ADAM17* is reported in abnormalities associated with heart, skin, lung and intestines [142], [143], [144]. Real time PCR confirmed array results of *ADAM17* (Table S4). *ADAM33* has been recently implicated as an asthma and bronchial hyperresponsiveness gene [145]. It is required for smooth muscle development in the lungs helps in airway wall “modeling”, and proper functioning of lungs throughout life [146], [147].

*CTSB* (Cathepsin B) also known as amyloid precursor protein secretase (*APPS*) was upregulated. It is involved in the proteolytic processing of amyloid precursor protein [148]. Felbor et al [149] reported deficiency of *CTSB* results in brain atrophy and loss of nerve cells in mice. *CTSC* (Cathepsin C) was downregulated in the L/C group and upregulated in the L3/C group. Loss of function mutations in *CTSC* gene are associated with tooth and skin abnormalities [150].

*NAALAD2* was upregulated while *PAPLN, RNF130, TMPRSS2, PGC, CPZ, FURIN* were downregulated. *CPZ* interacts with WNT proteins and may regulate embryonic development, however, its expression in adult tissues is less abundant [151]. *TPP2* and *SPPL2B* showed increased expression in L/C and decreased expression in L3/C. *PAPPA, GZMA, SERPINA1, QPCTL* transcripts were downregulated in L/C and upregulated in L3/C. Several hypothetical proteins (*FLJ10504, FLJ30679, FLJ90661, FLJ25179, DKFZp686L1818*) were differentially expressed.

### Cell Cycle, Cell Growth and Cell Proliferation

Fifteen transcripts having a role in cell cycle regulation, growth and proliferation were differentially expressed (Table S5). Four of the transcripts *SESN3, RAD1, GAS1* and *PARD6B* involved in cell cycle regulation were upregulated in both the groups. Cell growth factors, *INHBC* and *OGN* were induced in both the groups. *FGFR1OP* is a positive regulator of cell proliferation and showed increased expression. *KAZALD1, CDC20* and *CDKN2C* were downregulated.

Growth arrest specific gene 1 (*GAS1*) expression is positively required for postnatal cerebellum development. Mice lacking *GAS1* had significantly reduced cerebellar size compared to wild type mice [152]. Liu et al [152] proposed that *GAS1* perform dual roles in cell cycle arrest and in proliferation in a cell autonomous manner. *PARD6B* has a role in axonogenesis [153].

*INHBC* is a member of transforming growth factor-beta superfamily (TGF-beta) and is involved cell growth and differentiation [154], [155]. Osteoglycin (*OGN*) is also known as Mimecan and Osteoinductive factor (*OIF*). Mimecan is a member of small-leucine rich proteoglycan gene family and is a major component of cornea and other connective tissues [156], [157]. It has a role in bone formation , cornea development and regulation of collagen fibrillogenesis in corneal stroma [157], [158], [159]. *CDC20* regulates anaphase-promoting complex [160].

### Response to Stress

*MSRA, SOD2, GSTA3* and *GSR* genes were differentially expressed (Table S5). *MSRA* was upregulated in both the supplement groups. *SOD2* is downregulated in L/C and upregulated in L3/C. *GSR* is upregulated in the L/C and downregulated in the L3/C. *GSTA3* is downregulated in both the groups.

Oxidative damage to proteins by reactive oxygen species is associated with oxidative stress, aging, and age-related diseases [161], [162], [163]. *MSRA* is expressed in the retina, neurons and the nervous system [162]. Knock-outs of the *MSRA* gene in mice result in shortened life-spans both under normoxia and hyperoxia conditions [164]. *MSRA* also participates in the regulation of proteins [165]. *MSRA* plays an important role in neurodegenerative diseases like Alzheimer's and Parkinson's by reducing the effects of reactive oxygen species [163]. Overexpression of *MSRA* protects human fibroblasts against H2O2-mediated oxidative stress [166]. *SOD2* belongs to the iron/manganese superoxide dismutase family. It encodes a mitochondrial protein and helps in the elimination of reactive oxygen species generated within mitochondria [167]. In our study increased amount of DHA reduced the expression of glutathione-related proteins *GSR* and *GSTA3*.

### Kinases and Phosphatases

Phosphorylation and dephosphorylation of proteins control a multitude of cellular processes. Several proteins having kinase activity were altered (Table S5). Of note, transcripts involving *STK3, STK6, HINT3, TLK1, DRF1, GUCY2C* and *NEK1* were significantly upregulated with increasing DHA. A number of MAP kinases were downregulated in L3/C group, including *MAP4K1, MAPK12, MAP3K2* and *MAP3K3*. Other transcripts which showed significantly decreased expression were *CKM, LMTK2, NEK11, TNK1, BRD4* and *MGC4796*.

Transcripts having dephosphorylation activity, including *ACPL2, KIAA1240, PPP2R3A, PPP1R12B, PTPRG, PPP3CA* and *ACPP* were upregulated in L3/C group (Table S5). *MTMR2, PPP1R7, PTPRN2* and *HDHD3* were significantly downregulated with increasing DHA.

### Transcription Factors

Several transcription factors are differentially expressed by dietary LCPUFA (Table S5). Zinc finger proteins, Homeo box proteins and RNA Pol II transcription factors were among them. Several of the Zinc finger proteins were upregulated in L3/C, which include *ZNF611, ZNF584, ZNF81, ZNF273, ZNF547, MYNN, ZBTB11, PRDM7, JJAZ1, ZNF582, MLLT10, ZNF567, ZNF44, ZNF286, ZFX, NAB1, ZNF198, ZNF347* and *ZNF207*, while *PCGF2, ZBTB9, ZNF297, WHSCIL1, SALL4, ZNF589, ZFY, ZNF146, ZNF419* and *ZNF479* were repressed in L3/C group. Zinc finger proteins exhibit varied biological functions in eukaryotes including activation of transcription, protein folding, regulation of apoptosis, lipid binding etc [168]. Homeobox transcription factors, *TGIF2, PHTF1, OTP* and *HHEX* were induced whereas *PHOX2A, IRX1* and *MITF* were repressed in L3/C. RNA Pol II transcription factors (*BRCA1, TFCP2, CHD2, THRAP3, SMARCD2* and *NFE2L2*) showed increased expression in L3/C. However, transcripts for *UTF1, POU2F2, ELL, POLR2C, THRAP5, TGIF* and *GLIS1* showed decreased expression in L3/C. *SOX7* and *SOX12*, high mobility group (HMG) box proteins, were also differentially expressed. *ZNF611* array expression results were confirmed by real time PCR (Table S4).

### Receptor Activity

Transcripts performing receptor activities were differentially expressed (Table S5). While increasing levels of DHA were associated with decreased expression of *CD40, ITGB7, IL20RA, CD14, DOK3, MR1, BZRAP1, RARA, CD3D, IL1R1, MCP, HOMER3* transcripts, increased expression was detected for *FCGR2B, IL31RA, MRC2, SCUBE3, CR2, NCR2, CRLF2, SLAMF1, EGFR* and *KIR3DL2*. Interestingly, retinoic acid receptor α (*RARA*) activity was decreased in both the groups. *EGFR* expression levels were confirmed by QRT-PCR (Table S4).

### Ubiquitin Cycle

Twenty-five probe sets having a role in the ubiquitination process were differentially expressed (Table S5). Interestingly, five members of F-box protein family (*FBXL7, FBXL4, FBXL17, FBXW4* and *FBXW8*) showed increased expression in L3/C group. F-Box proteins participate in varied cellular processes such as signal transduction, development, regulation of transcription and transition of cell cycle. They contain protein-protein interaction domains and participate in phosphorylation-dependent ubiquitination [169], [170]. Proteins associated with anaphase-promoting complex (*CDC23* and *ANAPC1*) were downregulated in L3/C group.

### Others

Transcripts involved in 1) calcium ion binding (*MGC33630, UMODL1, FLJ25818, S100Z, MGC12458, ITSN2* and *PRRG3*), 2) zinc ion binding (*FGD5, ZFYVE28, PDLIM4, ZCCHC6, ZNF518* and *INSM2*), 3) ATP binding (*MMAA* and *C6orf102*), 4) GTP binding (*DOCK5, DOCK6, DOCK10, MFN1* and *GTP*), 5) nucleic acid binding (*IFIH1, C13orf10, DDX58, TNRC6C, RSN, ZCCHC5, DJ467N11.1, MGC24039* and *LOC124245*), 6) DNA binding (*KIAA1305, HP1-BP74, H2AFY, C17orf31, HIST1H2BD* and *HIST1H1E*) 7) protein binding (*ABTB1, MGC50721, RANBP9, STXBP4, BTBD5* and *KLHL14*) and 8) protein folding (*HSPB3, DNAJB12, FKBP11* and *TBCC*) were all differentially expressed. Also, several transcripts which play a role in RNA processing events were differentially expressed. *SFRS2IP*, LOC81691, *EXOSC2, SFPQ, SNRPN* and *SFRS5* showed increased expression, whereas, *NOL5A, RBM19, NCBP2* and *PHF5A* showed decreased expression with increasing DHA. Transcripts related to immune response are also differentially expressed. *HLA-DPB1, MX2* and *IGHG1* were upregulated and *PLUNC* was downregulated with increasing DHA.

Finally, 406 transcripts with no known gene ontology functions were differentially expressed (Table S5). Several of these transcripts were among the most differentially expressed, among these, *H63, LOC283403, FLJ13611, PARP6, C6orf111, C10orf67, TTTY8, C11orf1* and *PHAX* were upregulated, whereas transcripts for *CHRDL2, TSGA13, RP4-622L5*, MGC5391, *RNF126P1, FAM19A2* and *NOB1P* were repressed considerably.

### Ingenuity Network Analysis

We explored relationships among sets of genes using Ingenuity Systems network analysis. Out of 1108 differentially expressed probe sets in our data, 387 probe sets (34.93%) were found in the Ingenuity Pathway Analysis (IPA) knowledge database, and are labeled “focus” genes. Based on these focus genes, IPA generated 41 biological networks (Table S6). Among these 41 networks, 24 had scores of >8 and the top 2 networks with 35 genes had scores of 49. We focus here on the most significant network.

**Figure 1:**
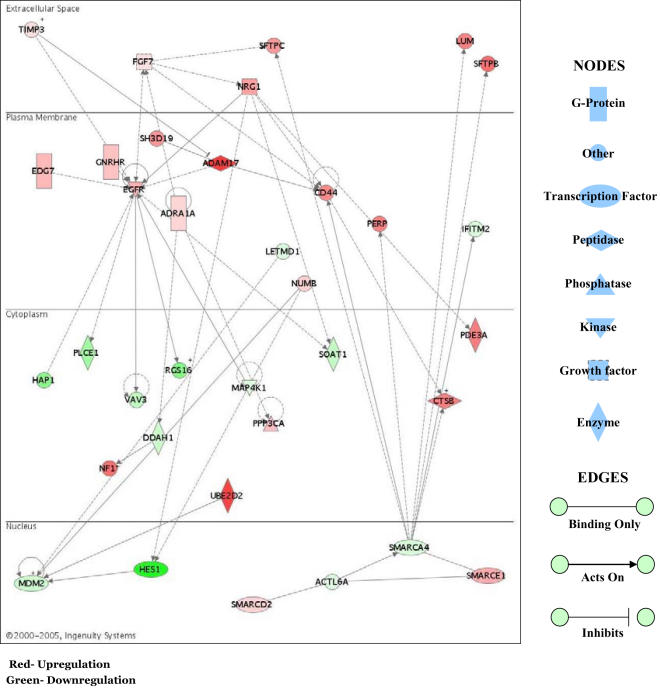
Ingenuity network analysis showing gene interactions generated from L3/C comparisons. Network is graphically represented as nodes (genes) and edges (the biological relationship between genes).

The top network identified by IPA is associated with nervous system development and function, cellular growth and proliferation (Figure 1). Epidermal growth factor receptor (*EGFR*) is the most outstanding interaction partner found within the network. *EGFR* interacts with *TIMP3, NRG1, ADAM17, EDG7* and *FGF7*; all are upregulated, and involved in neural or visual perception development. *EGFR* signaling is implicated in early events of epidermal, neural and eye development. Loss of *EGFR* signaling results in reduced brain size and loss of larval eye and optic lobe in *drosophila* [171]. *EGFR* expression is required for postnatal forebrain and astrocytes development in mice [172]. Functional pathway analysis conducted on this network using the IPA tool set identified three genes, *ADAM17, NUMB* and *HES1*, involved in the Notch signaling pathway which regulates nervous system and eye development [173], [174]. *ADAM17* and *NUMB* were upregulated while *HES1* was repressed in both the groups. This analysis suggests that LCPUFA influence many processes with influences that converge on *EGFR*.

LCPUFA are known to directly interact with nutrient sensitive transcription factors such as peroxisome proliferator-activated receptors (PPARs), liver×receptors, hepatic nuclear factor-4α, sterol regulatory binding proteins, retinoid×receptors and NF-КB. Upon ingestion, LCPUFA can elicit a transcriptional response within minutes[31], [175], [176], [177]. Microarray studies on LCPUFA-supplementing animals have identified several tissue-specific pathways regulated by LCPUFA, particularly involving the liver, adipose, and brain tissue transcriptome [26], [178], [179]. Using murine 11K Affymetrix oligoarrays, Berger et al [178] [180] showed increased hepatic expression of lipolytic and decreased expression of lipogenic genes. However, in the hippocampus brain region, increased expression of *HTR4* and decreased expression of *TTR* and *SIAT8E*, genes involved in the regulation of cognition and learning, as well as *POMC*, a gene associated with appetite control, was identified. The first paper published on the brain gene transcriptome with respect to LCPUFA supplementation by Kitajka et al. in 2002 [181] demonstrated that feeding fish oil (DHA 26.9%) to rats increased expression of genes involved in lipid metabolism (*SPTLC2, FPS*), energy metabolism (ATP synthase subunit d, ATP synthase H^+^, cytochromes, *IDH3G*), cytoskeleton (Actin related protein 2, *TUBA1*), signal transduction (Calmodulins, *SH3P4, RAB6B* small GTPase), receptors, ion channels and neurotransmission (Vasopressin V1b receptor, Somatostatin), synaptic plasticity (Synucleins) and regulatory proteins (protein phosphatases). In the same study, fish oil supplementation also significantly reduced the expression of phospholipase D and Transthyretin. In related work, Kitajka et al [26] , using rat cDNA microarrays with 3,200 spots, found results similar to those previously reported. Barcelo-Coblijn et al. [182] were the first to report moderation of age-induced changes in gene expression in rat brain as a result of diets rich in fish oil (DHA 11.2%). In this study, 2 month old rats showed increased expression of *SNCA* and *TTR*, however, 2-year old rats exhibited no significant changes. In addition, Puskas et al. [183] demonstrated that administration of omega-3 fatty acids from fish oil (5% EPA and 27% DHA; total fat content: 8%) for 4 weeks in 2 year old rats induced expression of transthyretin and mitochondrial creatine kinase and decreased expression of *HSP86, ApoC-I* and Makorin RING zinc-finger protein 2, genes in hippocampus brain region. Finally, Flachs et al [179] showed increased expression of genes for mitochondrial proteins in adipose tissue.

In comparison with previous brain transcriptome analyses, the present study employing the use of high-density Affymetrix oligoarrays (>54,000 ps) revealed genes differentially regulated by LCPUFA at ranges mimicking breastmilk. With the exception of *SPTLC2*, which we also found to be upregulated in the L/C and L3/C comparisons, none of the remaining, previously identified genes, were differentially expressed in our dataset. Many factors are likely to contribute to the observed differences in differentially expressed genes between our study and previous work. One likely source is the difference in dietary DHA/ARA, which is within the range of human and baboon breastmilk; previous studies used much higher amounts of DHA, from 11.2% to 27% [182], [183]. Also, interactions between levels of ARA and DHA supplied in our study add some complexity to the interpretation since the three treatments do not represent a strict dose response to DHA. However, our DHA and ARA come from sources that are routinely consumed by human infants in commercial infant formulas, and thus are directly relevant to that group. Despite lower levels of DHA/ARA, genes in our data set show subtle changes in expression. Moreover, the magnitude of these results is not surprising given the nutritional focus of the study, in which subtle, widespread shifts in transcription may have profound biological effects. Our data indicate that LCPUFA supplementation within the ranges of breastmilk will induce global changes in gene expression across numerous biological processes.

### Conclusions

The impact of DHA and ARA on infant baboons was both significant and widespread. We identified several novel differentially-expressed transcripts in 12-week old baboon cerebral cortexes modulated by dietary LCPUFA. The majority of probe sets showed subtle changes in gene transcription. In the cerebral cortex, we observed increased expression of mitochondrial proton carrier, *UCP2* (uncoupling protein 2) in both groups, but more in L3/C. *PLA2G6*, implicated in childhood neurodegeneration, was differentially expressed. *TIA1*, a silencer of the *COX2* gene translation is upregulated in L3/C. Increased expression was observed for *TIMM8A, NRG1, SEMA3D* and *NUMB*, genes involved in neural development. *LUM, EML2, TIMP3* and *TTC8* genes with roles in visual perception were upregulated. Hepatic nuclear factor-4α (*HNF4A*) showed decreased expression with increasing DHA. *RARA* was repressed in both the groups. A network involving 35 genes attributed to neural development and function was identified using Ingenuity pathway analysis, emphasizing *EGFR* as the most outstanding interaction partner in the network. In this network *EGFR* interacts with genes involved in neural or visual perception, *TIMP3, NRG1, ADAM17, EDG7* and *FGF7*. Although subtle, the upregulation of *NUMB* and downregulation of *HES1* in the Notch signaling pathway, not previously shown to interact with fatty acids, supports the involvement of LCPUFA, particularly DHA, in neural development. Interestingly, no known desaturases and only one elongase, LCPUFA biosynthetic enzymes, were differentially expressed in cerebral cortex. In a study of liver gene expression in preparation, fatty acid desaturases *SCD* and *FADS1* were significantly downregulated in liver, where we identified a multifunctional protein *TOB1* which is significantly upregulated.

These data represent the first comprehensive transcriptome analysis in primates and have identified widespread changes in cerebral cortex genes that are modulated by increases in DHA, induced by dietary means. Importantly, the range of DHA used here is within limits of human and primate breastmilks, the natural food for infants, and indicate that CNS gene expression responds to LCPUFA concentrations.

## Materials and Methods

Details of experimental design, animal characteristics, and tissue sampling are available elsewhere [38] and will be outlined briefly here.

### Animals and Diets

The animal phase took place at the Southwest Foundation for Biomedical Research (SFBR), San Antonio, TX, and was approved for animal care and research protocols from SFBR and Cornell University Institutional Animal Care and Use Committee (IACUC). Twelve baboon neonates born spontaneously around 182 days gestation were randomized into 3 groups (n = 4 per group). They were fed for 12 weeks on one of three formulas: C: Control (no DHA-ARA); L: 1×LCPUFA (0.33%DHA-0.67%ARA); L3: 3×LCPUFA (1.00%DHA-0.67%ARA). Formulas in color-coded cans were kindly provided by Mead-Johnson Nutritionals (Evansville, IN) in ready-to-feed form, 2 colors per treatment, so that investigators were masked to the treatments.

### Sampling and Array Hybridization

Twelve week old baboon neonates were anesthetized and euthanized at 84.4±1.1 days. Tissue collected from the precentral gyrus of the cerebral cortex was placed in RNALater according to vendor instructions and was used for the microarray analysis and validation of microarray results.

Microarray studies utilizing baboon samples with human oligonucleotide arrays have been successfully carried out previously[184], [185]. Cerebral cortex global messenger RNA in the three groups was analyzed using Affymetrix Genechip™ HG-U133 Plus 2.0 arrays <http://www.affymetrix.com/products/arrays/specific/hgu133plus.affx>. The HG-U133 Plus 2.0 has >54,000 probe sets representing 47,000 transcripts and variants, including 38,500 well-characterized human genes. One hybridization was performed for each animal (12 chips total). RNA preparations and array hybridizations were processed at Genome Explorations, Memphis, TN <http://www.genome-explorations.com>. The completed raw data sets were downloaded from the Genome Explorations secure ftp servers.

### Microarray Data Analysis

Raw data (.CEL files) were uploaded into Iobion's Gene Traffic MULTI 3.2 (Iobion Informatics, La Jolla, CA, USA) and analyzed by using the robust multi-array analysis (RMA) method. In general, RMA performs three operations specific to Affymetrix GeneChip arrays: global background normalization, normalization across all of the selected hybridizations, and log2 transformation of perfect match oligonucleotide probe values [186]. Statistical analysis using the significance analysis tool set in Gene Traffic was utilized to perform Multiclass ANOVA on all probe level normalized data. Pairwise comparisons were made between C vs L and C vs L3 and all probe set comparisons reaching *P* <0.05 were included in the analysis. Gene lists of differentially expressed probe sets were generated from this output for functional analysis.

### Bioinformatics analysis

Expression data was annotated using NIH DAVID <http://apps1.niaid.nih.gov/david> [187] and NetAffx <http://www.affymetrix.com/analysis/index.affx>. Genes were grouped into functional categories and pathways based on the Gene Ontology Consortium <http://www.geneontology.org>, Kyoto Encyclopedia of Genes and Genomes (KEGG) pathway Database <http://www.genome.jp/kegg/pathway.html> and <BioCarta <http://www.biocarta.com/>. Data presented in this manuscript is accessible through GEO Series accession number GSE6519 (GEO, http://www.ncbi.nlm.nih.gov/geo/).

### RNA Isolation and RT PCR

RT PCR was conducted on nine genes to confirm the results of the array analysis. Total RNA from 30 mg samples of baboon cerebral cortex brain tissue homogenates was extracted using the RNeasy Mini kit (Qiagen, Valencia, CA). Each RNA preparation was treated with DNase I according to the manufacturer's instructions. The yield of total RNA was assessed by 260 nm UV absorption. The quality of RNA was analyzed by 260/280 nm ratios of the samples and by agarose gel electrophoresis to verify RNA integrity.

One microgram total RNA from each group (C, L, L3) was reverse-transcribed into first strand cDNA using the iScript cDNA synthesis kit (Bio-Rad, Hercules, CA). The iScript reverse transcriptase is a modified MMLV-derived reverse transcriptase and the iScript reaction mix contains both oligo(dT) and random primers. The generated first strand cDNA is stored at −20°C until used.

Quantitative real-time PCR using SYBR green and TaqMan assay methods was used to verify the differential expression of selected genes that were upregulated in L3/C comparison. All the primers were gene-specific and generated from human sequences <www.ensembl.org>. PCR primers were designed with PrimerQuest software (IDT, Coralville, IA) and ordered from Integrated DNA Technologies (IDT, Coralville, IA). Initially primers were tested by polymerase chain reactions with baboon cerebral cortex brain cDNA as template in a 30 µl reaction volume using Eppendorf gradient thermal cycler (Eppendorf), with 1 µm of each primer, 0.25 mM each of dNTPs, 3 µl of 10×PCR buffer (Perkin-Elmer Life Sciences, Foster City, CA, USA), 1.5 mM MgCl_2_ and 1.5 U *Taq* polymerase (Ampli *Taq* II; Perkin-Elmer Life Sciences). Thermal cycling conditions were: initial denaturation at 95°C for 5 min followed by 25–35 cycles of denaturation at 95°C for 30 s, annealing at 60°C for 1 min and extension at 72°C for 1 min, with a final extension at 72°C for 2 min. PCR products were separated by electrophoresis on 2% agarose gel stained with ethidium bromide and bands of appropriate sizes were obtained. The PCR products of *LUM, TIMM8A, UCP2, ß-ACTIN, ADAM17* and *ATP8B1* were sequenced and deposited with GenBank (Acc Numbers: DQ779570, DQ779571, DQ779572, DQ779573, DQ779574 and DQ779575, respectively).

Initially standardized primers for genes (*ATP8B1, ADAM17, NF1, FZD3, ZNF611, UCP2, EGFR* and control *ß-ACTIN*) were used for SYBR green real time PCR assay (Power SYBR Green PCR Master Mix, Applied Biosystems, Foster City, CA). We used the baboon *LUM, TIMM8A* and *ß-ACTIN* sequences to design TaqMan Assay (Assay by Design; <www.appliedbiosystems.com>). The selected gene symbols, primer pairs and probe details are depicted in Table S3. Quantitative real time PCR reactions were done with the Applied Biosystems Prism 7300/7500 real time PCR system (Applied Biosystems, Foster City, CA). After 2 minutes of UNG activation at 50°C, initial denaturation at 95°C was carried out for 10 minutes, the cycling conditions of 40 cycles consisted of denaturation at 95°C for 15 seconds, annealing at 60°C for 30 seconds, and elongation at 72°C for 1 minute. For SYBR green method UNG activation step is eliminated. All reactions were done in triplicate and *ß-ACTIN* was used as the reference gene. Relative quantification was performed by using comparative CT method (ABI Relative Quantification Chemistry guide # 4347824).

### Network Analysis

We used a new web-delivered bioinformatics tool set, Ingenuity pathway analysis (IPA 3.0) <http://www.ingenuity.com>, to identify functional networks influenced by our dietary treatments. IPA is a knowledge database generated from the peer-reviewed scientific publications that enables discovery, visualization and exploration of functional biological networks in gene expression data and delineates the functions most significant to those networks. The 1108 differentially expressed probe sets identified by microarray data, as discussed below, were used for network analyses. Affymetrix probe set ID's were uploaded into IPA and queried against all other genes stored in the IPA knowledge database to generate a set of networks having up to 35 genes. Each Affymetrix probe set ID was mapped to its corresponding gene identifier in the IPA knowledge database. Probe sets representing genes having direct interactions with genes in the IPA knowledge database are called “focus” genes, which were then used as a starting point for generating functional networks. Each generated network is assigned a score according to the number of differentially regulated focus genes in our dataset. These scores are derived from negative logarithm of the *P* indicative of the likelihood that focus genes found together in a network due to random chance. Scores of 4 or higher have 99.9% confidence level of significance as defined in detail elsewhere [188].

## Supporting Information

Table S1A Genes with known function upregulated by L3 (1.00%DHA-0.67%ARA) in Brain
(0.16 MB XLS)

Table S1B Genes with known function downregulated by L3 (1.00%DHA-0.67%ARA) in Brain
(0.18 MB XLS)

Table S1C Genes without known function upregulated by L3 (1.00%DHA-0.67%ARA) in Brain
(0.08 MB XLS)

Table S1D Genes without known function downregulated by L3 (1.00%DHA-0.67%ARA) in Brain
(0.07 MB XLS)

Table S2 Probe sets showing ≥1.4 fold changes in gene expression
(0.02 MB XLS)

Table S3 Primers and Probe Sequences
(0.02 MB XLS)

Table S4 Comparison of microarray versus QRT-PCR gene expression values (Fold-changes)
(0.01 MB XLS)

Table S5 Classification According to Gene Ontology Functions for Brain
(0.28 MB XLS)

Table S6 Ingenuity functional network analysis
(0.07 MB XLS)
